# One-year clinical outcomes of MR-guided stereotactic body radiation therapy with rectal spacer for patients with localized prostate cancer

**DOI:** 10.1007/s00345-024-04784-x

**Published:** 2024-02-23

**Authors:** Darren M. C. Poon, Jing Yuan, Oi Lei Wong, Bin Yang, Mei Yan Tse, Ka Ki Lau, Sin Ting Chiu, Peter Ka-Fung Chiu, Chi Fai Ng, Ka Lun Chui, Yiu Ming Kwong, Wai Kit Ma, Kin Yin Cheung, George Chiu, Siu Ki Yu

**Affiliations:** 1 Comprehensive Oncology Centre, 11/F, HKSH Eastern Building, 3 Tung Wong Roade Road, Shau Kei Wan, Hong Kong SAR; 2 Research Department, Hong Kong Sanatorium and Hospital, Happy Valley, Hong Kong, Hong Kong SAR; 3 Medical Physics Department, Hong Kong Sanatorium and Hospital, Happy Valley, Hong Kong, Hong Kong SAR; 4 Department of Radiotherapy, Hong Kong Sanatorium and Hospital, Happy Valley, Hong Kong, Hong Kong SAR; 5 SH Ho Urology Centre, Department of Surgery, The Chinese University of Hong Kong, Shatin, New Territories Hong Kong SAR; 6 Urology Centre, Hong Kong Sanatorium and Hospital, Happy Valley, Hong Kong, Hong Kong SAR; 7 Hong Kong Urology Clinic, Hong Kong, Hong Kong SAR

**Keywords:** Prostate cancer (PC), Rectal spacer, Magnetic resonance-guided stereotactic body radiation therapy (MRgSBRT), Toxicity, Patient-reported outcomes (PROs)

## Abstract

**Background and purpose:**

This prospective study aimed to investigate adaptive magnetic resonance (MR)-guided stereotactic body radiation therapy (MRgSBRT) with rectal spacer for localized prostate cancer (PC) and report 1-year clinical outcomes.

**Materials and methods:**

Thirty-four consecutive patients with low- to high-risk localized PC that underwent 5-fraction adaptive MRgSBRT with rectal spacer were enrolled. The dosimetric comparison was performed on a risk- and age-matched cohort treated with MRgSBRT but without a spacer at a similar timepoint. Clinician-reported outcomes were based on Common Terminology Criteria for Adverse Events. Patient-reported outcomes were based on the Expanded Prostate Cancer Index Composite (EPIC) questionnaire at baseline, acute (1–3 months), subacute (4–12 months), and late (> 12 months) phases.

**Results:**

The median follow-up was 390 days (range 28–823) and the median age was 70 years (range 58–82). One patient experienced rectal bleeding soon after spacer insertion that subsided before MRgSBRT. The median distance between the midline of the prostate midgland and the rectum after spacer insertion measured 7.8 mm (range 2.6–15.3), and the median length of the spacer was 45.9 mm (range 16.8–62.9) based on T2-weighted MR imaging. The use of spacer resulted in significant improvements in target coverage (V100% > 95% = 98.6% [range 93.4–99.8] for spacer vs. 97.8% [range 69.6–99.7] for non-spacer) and rectal sparing (V95% < 3 cc = 0.7 cc [range 0–4.6] for spacer vs. 4.9 cc [range 0–12.5] for non-spacer). Nine patients (26.5%) experienced grade 1 gastrointestinal toxicities, and no grade ≥ 2 toxicities were observed. During the 1-year follow-up period, EPIC scores for the bowel domain remained stable and were the highest among all other domains.

**Conclusions:**

MRgSBRT with rectal spacer for localized PC showed exceptional tolerability with minimal gastrointestinal toxicities and satisfactory patient-reported outcomes. Improvements in dosimetry, rectal sparing, and target coverage were achieved with a rectal spacer. Randomized trials are warranted for further validation.

**Supplementary Information:**

The online version contains supplementary material available at 10.1007/s00345-024-04784-x.

## Introduction

External beam radiation therapy (RT) is one of the standards of care for localized prostate cancer [1]. With recent advances in intensity modulation and image guidance, hypofractionated RT has been widely utilized on the basis of its noninferior results compared to conventional fractionated RT [2]. Ultra-hypofractionated RT, also known as stereotactic body RT (SBRT), entailing 5–6 fractions of radiation, has become increasingly popular [3], with clinical studies demonstrating its feasibility and safety in prostate cancer and promising results in terms of clinical outcomes and quality of life (QOL) [4, 5]. However, because of the increased dose per fraction in SBRT, there is concern about the potential elevation of rectal toxicities and associated negative impacts on QOL [4].

To alleviate the potential increased risk of rectal toxicity due to the substantially higher radiation dose to the rectal wall, especially with prostate SBRT, injectable rectal spacers have been developed to increase the distance between the anterior rectum and the prostate [6]. The use of rectal spacers has been investigated in clinical studies, and promising preliminary results have been reported in conventional fractionated RT [7–9], hypofractionated RT, and SBRT [10–12].

In recent years, SBRT with online guidance with magnetic resonance imaging (MRgSBRT) conducted on integrated MR imaging (MRI) and linear accelerator (MR-LINAC) systems have been actively investigated to improve oncological outcomes of prostate RT and QOL by taking advantage of the superior soft tissue contrast of MRI and daily plan adaptation based on anatomical changes and real-time motion management capability. Preliminary results of prostate MRgSBRT are promising [13–16]. For instance, the randomized phase 3 MIRAGE trial revealed that, compared with computed tomography (CT)-guided SBRT, MR-guided SBRT was associated with lower incidences of acute grade ≥ 2 genitourinary (GU) and gastrointestinal (GI) toxicities in patients with localized prostate cancer. However, longer-term data are still pending [16].

The potential rectal-protective benefit with rectal spacers may be further enhanced in the setting of MRgSBRT because rectal spacers are better visualized as hyper-intensity on T2-weighted MRI than on CT. Several studies have shown similar dosimetric advantages of rectal hydrogel spacers in non-MR-guided RT and MRgSBRT plans to rectal walls and other organs-at-risk (OARs) [17, 18]. The asymmetrical placement of the rectal spacer is detectable, and its potential negative dosimetric impact can be addressed on online adaptive MR images [19]. Recently, Alongi et al. also reported the first preliminary patient-reported outcomes (PROs) of prostate cancer patients receiving hydrogel spacers at the end of their MRgSBRT treatment [20]. Their results suggest that both rectal sparing and target coverage favor the application of the rectal spacer.

No studies have reported the acute and long-term toxicities and PROs of prostate cancer with rectal spacers after MRgSBRT treatment. Thus, the major aim of this study was to prospectively investigate the clinical use of rectal spacer for adaptive MRgSBRT in localized prostate cancer patients and to report the PROs and clinician-reported outcomes (CROs) up to a median follow-up of 1 year.

## Material and methods

### Patient selection

This prospective observational study was approved by our hospital research ethics committee (REC-2021-28). All prostate cancer patients who were willing to receive insertion of rectal spacer prior to adaptive MRgSBRT on a 1.5-Tesla (T) MR-LINAC were eligible for enrollment. Written consent was obtained from each participant.

Inclusion criteria were: age > 18 years; histologically proven prostate adenocarcinoma and no nodal or distant metastasis identified by MRI and/or prostate-specific membrane antigen (PSMA) positron emission tomography (PET); no history of other cancers; no previous prostate surgery other than transurethral resection of the prostate (> 6 months before MRgSBRT); and Eastern Cooperative Oncology Group (ECOG) Performance Status < 2. Exclusion criteria were: previous prostate/pelvic irradiation or prostate surgery; history of other cancers; MRI contraindication; MRI- and/or PSMA-PET-identified nodal or distant metastasis; ECOG Performance Status ≥ 2; inflammatory bowel disease; inability to obtain written informed consent; or patient non-completion of MRgSBRT by the lock date (31st January 2023) or dropout of the study within 15 days after MRgSBRT treatment without submission of PRO data.

### Rectal spacer insertion and simulation scans

The option of rectal spacer was discussed with all localized prostate cancer patients who were planned for 1.5 T MRgSBRT from August 2020 onward. Polyethylene glycol (PEG)-based hydrogel (SpaceOAR, Boston Scientific, MA, USA) and hyaluronic acid (HA) (Barrigel, Palette Life Sciences, Stockholm, Sweden) rectal spacers were allowed and were selected at the discretion of the urologist. The rectal spacer (~ 10 mL volume) was inserted into the perirectal space posterior to rectoprostatic fascia with ultrasound guidance under local/general anesthesia by a urologist, following common procedures described elsewhere [6], at least 10 days prior to the simulation scans.

After spacer insertion, CT and MRI simulations were conducted with a full bladder and empty rectum, both in the treatment position on the same day, using a dedicated CT simulator and a 1.5 T MRI simulator, normally within 1–2 weeks prior to MRgSBRT. A rectal balloon with 40–50 mL saline inflation was also used. A three-dimensional T2-weighted turbo spin echo (3D-T2W-TSE) pulse sequence with identical imaging parameters to daily online MRI on the MR-LINAC was scanned in simulation, along with other sequences, as per clinical purposes [14, 15].

### Treatment planning

The detailed treatment planning procedure was described in our previous studies [14, 15]. Generally, a 36.25- or 40-Gy dose in 5 fractions (delivered twice per week) was prescribed to the clinical target volume (CTV) in low- or intermediate/high-risk patients, respectively. Simultaneous dose boosting with 40 or 42.5 Gy in 5 fractions was optionally prescribed to the MR-visible dominant intraprostatic lesions (DILs). Concomitant 25 Gy in 5 fractions was also prescribed to the whole pelvis in high-risk patients. Androgen-deprivation therapy (ADT) with a luteinizing hormone-releasing hormone agonist or antagonist was generally planned for durations of 6 months and 18–36 months for unfavorable intermediate-risk and high-risk patients, respectively. The prescription of ADT for favorable intermediate-risk patients was at the discretion of the physician.

The increase in prostate–rectum distance along the midline of the prostate mid-gland and spacer width were measured on the transversal T2-weighted planning MR images. The inserted spacer length was measured on T2-weighted planning MR images in sagittal view. To evaluate the dosimetric effect of rectal spacer insertion on treatment planning, the spacer cohort (*n* = 34) was retrospectively compared to a risk- and age-matched non-spacer cohort (*n* = 34). In this non-spacer cohort, all patients were also treated with MRgSBRT but without a spacer at a similar timepoint. The matching was designed to ensure that the spacer and non-spacer cohorts had no significant differences in medical and surgical comorbidity, age, and risk level. The medical and surgical comorbidity factors included diabetes, hypertension, ischemic heart disease, hemorrhoid, prior abdominal or pelvic surgery, and the use of anti-coagulants.

### Treatment delivery and adaptation

At each fraction, a daily 3D-T2W-TSE MRI scan was performed to obtain on-the-date anatomy information [21]. Online plan adaptation was conducted using either adapt-to-position (ATP), which was prioritized to maximize workflow efficiency, or adapt-to-shape (ATS), which was adopted to handle substantial anatomical changes [22]. The necessity of ATS was determined using institutional criteria and the attending oncologist’s experience [22]. If ATS was adopted, the attending oncologist adapted the contours of the CTV, rectum, bladder, spacer, and bowel via manual contouring and/or deformable registration, if necessary. Plan re-optimization on the adjusted contours was applied to achieve all planned dosimetric criteria. Optimal target coverage was prioritized to constrain doses to OARs. An online patient-specific quality assurance was also performed, followed by another MRI scan to confirm patient position consistency. Immediately after positional confirmation, the dose was delivered as per the adapted plan.

### Patient follow-up and outcome measurements

Complications and side effects associated with spacer insertion were recorded by the urologist and radiation oncologist. If severe side effects occurred after spacer insertion, simulation and planning were postponed until the patient recovered.

Follow-up examinations by the radiation oncologist were scheduled during (3rd fraction) and at the completion date (5th fraction) of MRgSBRT, at 1 month, every 3 months in the first year, and every 6 months thereafter until 11th January 2023, and were categorized as intratreatment (during MRgSBRT), acute (1–3 months), subacute (3–12 months), and late (> 12 months) phases. CROs were mainly assessed from GI and GU toxicities using the Common Terminology Criteria for Adverse Events (CTCAE) scale v 5.0. PROs were assessed by the QOL domains of the Expanded Prostate Cancer Index Composite (EPIC) questionnaire [23]. Patients were requested to complete an EPIC questionnaire at baseline (prior to spacer insertion), 1 month, every 3 months in the first year, and every 6 months thereafter. The patient data were locked on 31st January 2023.

### Statistical analysis

All statistical analyses were performed using R v1.2 (RStudio, Boston, MA, USA). Each patient’s follow-up duration was defined as the time interval (in months) between the last MRgRT treatment fraction and the last clinical follow-up visit. The median follow-up in the spacer cohort was calculated using the reverse Kaplan–Meier method. Descriptive statistics are expressed as medians with ranges. If a patient submitted more than one EPIC questionnaire at a predefined phase, the non-blank items were averaged to adjust for repeated measurements in the patient-reported QOL analysis. Dosimetric parameters between the spacer and non-spacer cohorts were compared using the Wilcoxon signed-rank test. The Kruskal–Wallis *H* test or the Mann–Whitney *U* test was used to compare the longitudinal QOL at different follow-up timepoints, where appropriate. The significant level was set at 0.05, and the Bonferroni correction was applied for multiple statistical tests.

## Results

### Patient selection and baseline characteristics

Thirty-eight biopsy-confirmed localized prostate cancer patients with rectal spacer insertion were eligible. Two were excluded because they had not finished MRgSBRT by the lock date. Two were excluded because they dropped out within 15 days after MRgSBRT treatment and did not provide PRO data. Ultimately, 34 patients fulfilled all inclusion criteria. Baseline patient characteristics are listed in Table 1.

**Table 1:** Patient characteristics at baseline (*n* = 34)

	Number of patients	Percentage
Median age, years (range)	70 (58–82)	
*ECOG performance status*		
0	31	91.1%
1	3	8.9%
*Clinical T stage (by multi-parametric MRI and biopsy)*		
1	0	0
2	26	76.5%
3	8	23.5%
*PSA* (*ng/mL*)		
Median (range)	11 (5–86)	
< 10	15	44.1%
10–20	11	32.4%
> 20	8	23.5%
*Gleason score*		
3 + 3	10	29.4%
3 + 4	11	32.4%
4 + 3	4	11.8%
4 + 4	4	11.8%
> 8	5	14.7%
*NCCN risk classification*		
Low	4	11.8%
Favorable intermediate	12	35.3%
Unfavorable intermediate	3	8.9%
High	12	35.3%
Very high	3	8.9%
*ADT*		
No	8	23.5%
Yes	26	76.5%

*NCCN* National comprehensive cancer network

### Spacer insertion, dosimetrics, and MRgSBRT treatment delivery

**Fig. 1:**
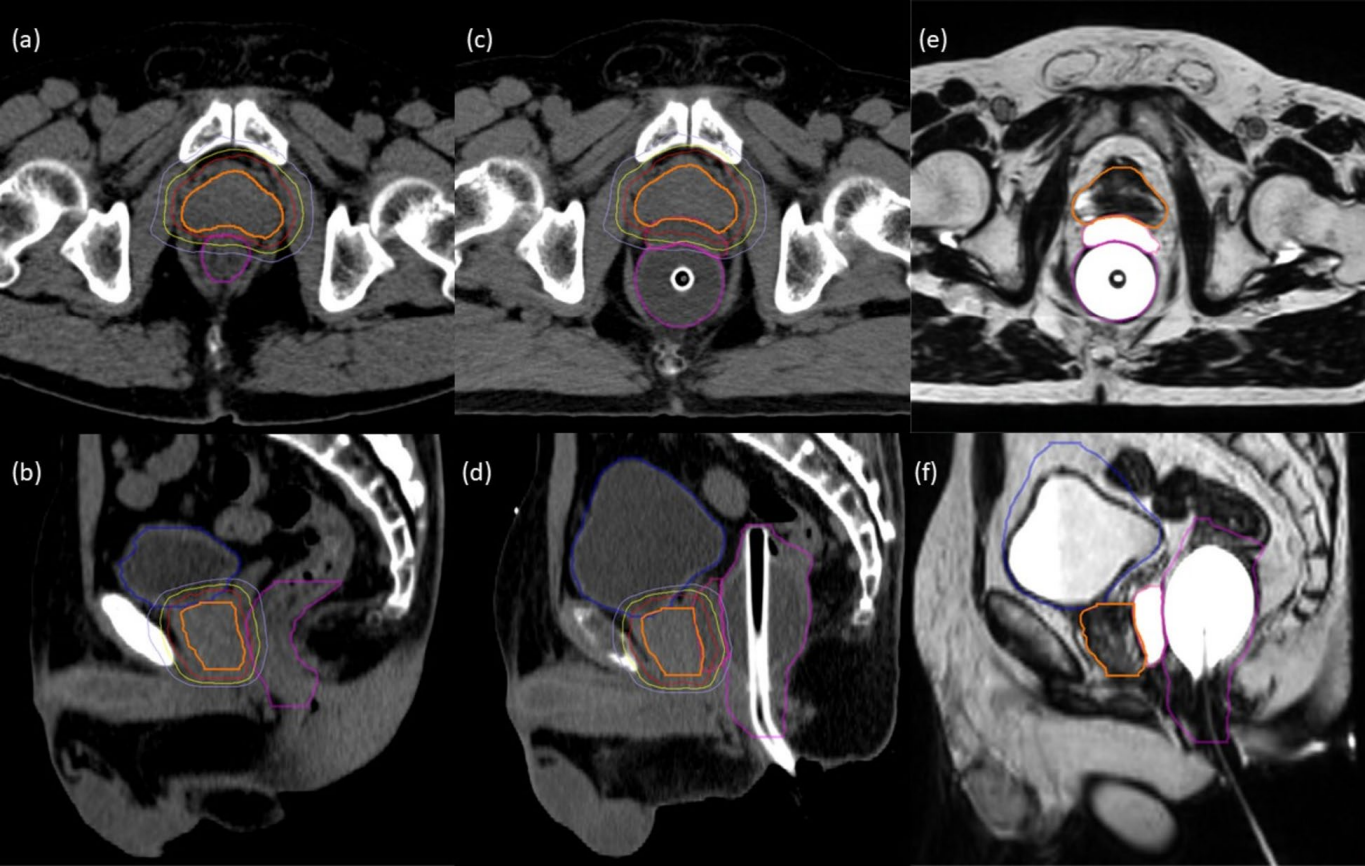
Transverse (**a**, **c**, **e**) and sagittal (**b**, **d**, **f**) CT and T2-weighted planning MR images in a patient before and after rectal spacer injection. (**a**, **b**) CT images acquired before spacer injection. **c**, **d** Planning CT images acquired after spacer injection. **e**, **f** Corresponding T2-weighted planning MR images. Thick lines correspond to contours of the CTV (orange), rectum (magenta), bladder (blue), and spacer (pink); thin lines correspond to 100% (red), 90% (yellow), and 75% (light blue) isodoses of the prescription dose (reference treatment plan) based on the post-spacer CT images also illustrated here

Procedural complications following rectal spacer insertion occurred in an 80-year-old patient who experienced rectal bleeding about 1 week after spacer insertion. The rectal bleeding completely subsided before the initiation of MRgSBRT. HA and hydrogel spacers were implanted in 31 and 3 patients, respectively. For all patients, the inserted spacers were clearly visible on T2W planning MRI and daily online MRI. The median distance increase between the prostate midgland and rectum (at midline) after spacer insertion was 7.8 mm (range 2.6–15.3), with a median spacer width of 36.2 mm (range 14.1–45.7). The median spacer length was 45.9 mm (range 16.8–62.9). A summary of the dosimetric parameters in the spacer and non-spacer cohorts is provided in Supplementary Table 1. Regarding target coverage, V100% of the planning target volume (PTV) was significantly higher in the spacer cohort (98.6%; range 93.4–99.8) than in the non-spacer cohort (97.8%; range 69.6–99.7; *p* = 0.03). Dose exposure to the rectum in terms of V105%, V95%, V90%, and V80% was significantly smaller with spacer insertion than without (*p* < 0.001). The median values of rectum V95% did not meet the dose constraints in the non-spacer cohort. Insignificant differences in dosimetric parameters were observed in other OARs. Figure 1 shows CT and T2-weighted planning MR images before and after HA rectal spacer insertion, and the prescribed isodose lines from the reference treatment plan based on post-spacer CT images.

In total, 170 (34 × 5) MRgSBRT fractions, comprising 155 ATP (91.2%) and 15 ATS (8.8%), were successfully delivered, with average fraction durations of ~ 60 min and ~ 90 min for ATP and ATS, respectively.

### Patient follow-up and clinical outcome

Post-MRgSBRT, patients were followed for a median of 390 days (range 28–823 days). There were 34, 30, and 19 patients assessed at the acute (1–3 months), subacute (3–12 months), and late (> 12 months) phases, respectively.

CRO measurements of GI and GU toxicities are summarized in Table 2. Both GI and GU adverse events were generally mild (mostly Grade 1 [G1] toxicities). Thirteen G1 GI adverse events occurred in 26.5% of patients (9/34), including 3 rectal hemorrhages (1 in the acute phase and 2 in the subacute phase) and 1 rectal pain (intratreatment phase). All were resolved at subsequent follow-ups. No Grade 2 (G2) or more severe GI toxicity was observed in any phase of follow-up.

**Table 2:** Distribution of clinician-reported GI and GU toxicities (grade [G] ≥ 1) based on the CTCAE 5.0 in the study patients (*n* = 34)

Follow-up phase	Intratreatment		Acute		Subacute		Late	
Toxicity grade (CTCAE v. 5.0)	G1	G2	G1	G2	G1	G2	G1	G2
*GI Toxicity*								
Abdominal pain	1	0	0	0	0	0	0	0
Bloating	0	0	0	0	0	0	0	0
Constipation	2	0	0	0	2	0	0	0
Diarrhea	2	0	0	0	0	0	0	0
Fecal incontinence	0	0	0	0	0	0	0	0
Nausea	0	0	0	0	0	0	0	0
Proctitis	0	0	3	0	0	0	0	0
Rectal hemorrhage	0	0	1	0	2	0	0	0
Rectal pain	1	0	0	0	0	0	0	0
*GU Toxicity*								
Urinary frequency	20	1	23	0	18	0	8	0
Urinary incontinence	1	0	4	0	4	0	1	0
Urinary retention	3	0	1	0	1	0	1	0
Urinary tract pain	9	1	4	0	2	0	2	0
Urinary urgency	4	0	4	0	1	0	0	0

**Table 3:** Patient-reported QOL metrics based on the EPIC questionnaire

	Timepoint				
	Baseline	Acute (1–3 months)	Subacute (4–12 months)	Late (> 12 months)	*P* value*
Patients (*n*)	21	25	24	13	NA
*Median domain summary scores [range]*					
Urinary	89.6 [60.6, 100.0]	83.3 [40.3, 97.9]	85.0 [53.5, 100.00]	84.0 [62.3, 100.0]	0.59
Bowel	94.6 [78.5, 100.0]	90.2 [3.9, 100.0]	95.8 [62.5, 100.0]	94.4 [70.2, 100.0]	0.63
Sexual	48.7 [19.2, 80.8]	30.8 [0, 68.2]	23.1 [0, 60.9]	20.7 [3.9, 60.6]	0.36
Hormonal	96.6 [68.2, 100.0]	85.5 [31.8, 100.0]	82.0 [53.8, 100.0]	88.6 [52.3, 100.0]	0.59
*Median gastrointestinal subcategorical scores [range]*					
Bowel function	96.4 [67.9, 100.0]	85.7 [25.0, 100.0]	96.1 [60.7, 100.0]	94.6 [60.7, 100.0]	0.65
Bowel bother	96.4 [67.9, 100.0]	92.9 [50.0, 100.0]	96.1 [39.3, 100.0]	96.0 [40.5, 100.0]	0.57

^*^Based on a non-parametric Kruskal–Wallis test. *NA* not applicable

GU toxicities were more common: 117 G1 and 2 G2 GU adverse events in 32 patients (94.1%) throughout the follow-up. The most common GU toxicities were urinary frequency (*n* = 70), followed by urinary tract pain (*n* = 18) and urinary incontinence (*n* = 10). Two G2 toxicities (1 urinary frequency and 1 urinary tract pain) occurred in a single patient during MRgSBRT and were resolved at subsequent follow-ups. Besides GI and GU toxicities, G1 fatigue was observed in 3 patients in the acute and/or subacute phases.

**Fig. 2:**
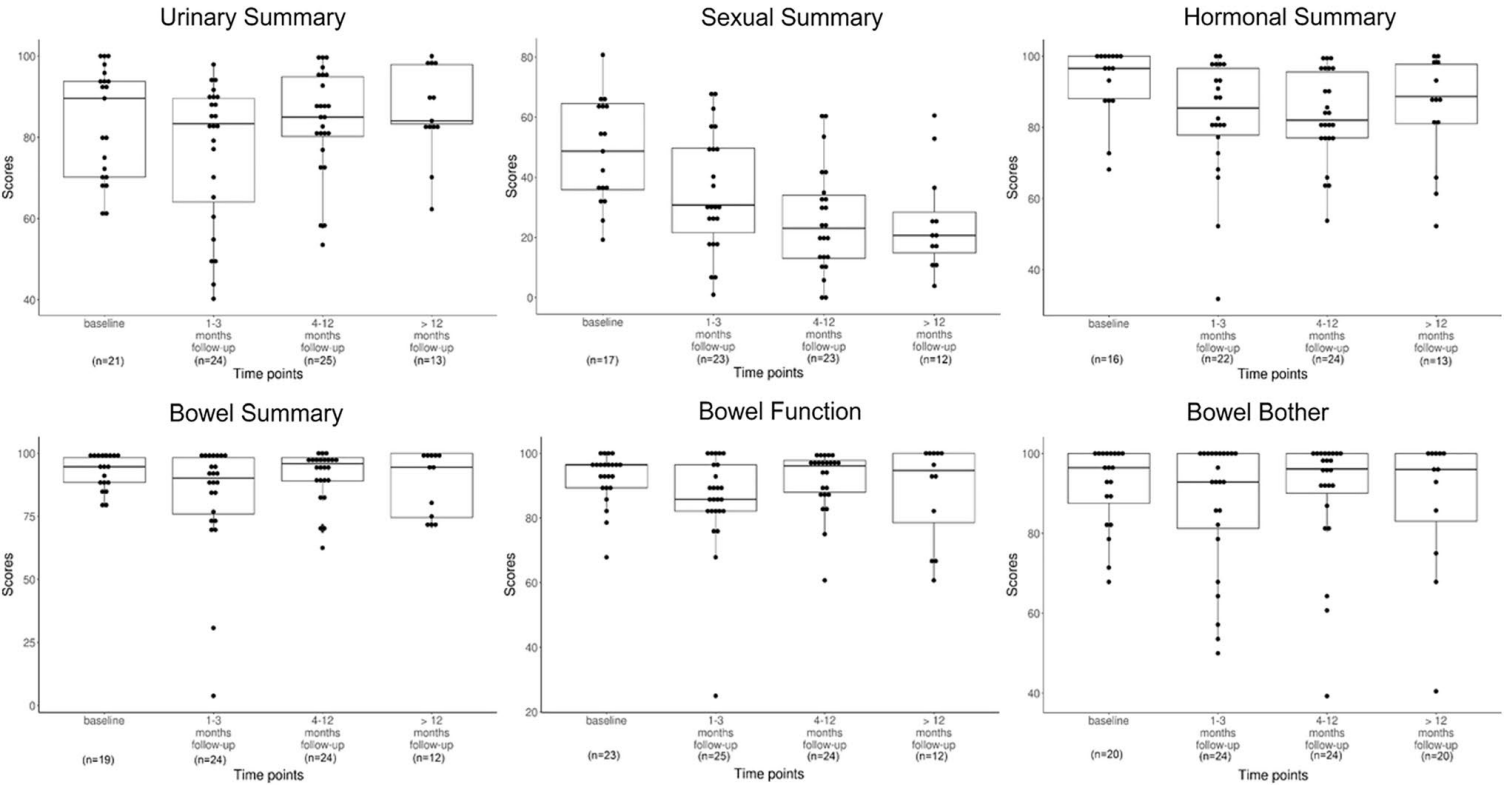
Longitudinal changes in EPIC composite domain summary scores and GI subcategorical scores during the follow-up period

Thirty out of 34 patients (overall response rate: 88.2%) submitted at least one EPIC questionnaire at baseline and different phases of follow-up. Twenty-one, 24, 24, and 13 patients submitted at least one EPIC questionnaire at baseline, acute, subacute, and late phases, respectively, corresponding to response rates of 61.8% (21/34), 70.6% (24/34), 80.0% (24/30), and 68.4% (13/19) at each phase. Twelve patients (35.3%) completed all EPIC questionnaires at the baseline and three follow-up phases. Patient-reported QOL scores based on the EPIC questionnaire are illustrated in Table 3 and Fig. 2. The four domain summary scores did not change significantly throughout the entire follow-up period (all *p* > 0.05). Notably, the bowel domain scores remained nearly constant and were the highest among all four domains at each follow-up phase. In the subcategories of bowel function and bowel bother, high QOL scores were generally maintained throughout the follow-up. A slight drop in bowel function was observed in the acute phase, but was fully recovered in the subacute phase, and was concordant with the clinician-reported GI toxicities that occurred in the intratreatment and acute phases.

## Discussion

Physical separation of the closely positioned posterior prostate and anterior wall of the rectum using injectable rectal spacers has been increasingly common to reduce radiation-induced rectal toxicity prior to definitive RT of localized prostate cancers. The low risk of rectal spacer placement and a low complication rate post-spacer placement are strongly supported by clinical data [23].

The efficacy of hydrogel spacers to lower the risk of clinically important GI complications of localized prostate cancer SBRT in 11 studies (780 patients) was recently summarized [24]. However, the corresponding evidence for HA spacers rather than hydrogel spacers in post-SBRT clinical outcomes is relatively sparse. Recently, Repka et al. outlined a framework and rationale for the utilization of rectal spacers in unfavorable-risk prostate cancer with dose-escalated SBRT [25].

In this study, we explored the use of HA spacers (majority) in localized prostate cancer MRgSBRT conducted on a high-field MR-LINAC and reported the 1-year clinical outcome in terms of PRO and CRO. As a comparison, in the study by Alongi et al.[20], they only reported preliminary PROs of prostate cancer patients with hydrogel spacers immediately at the end of their 1.5 T MRgSBRT, without providing longer follow-up data. Although the MIRAGE study had 44% of patients with hydrogel rectal spacer, the outcomes of patients with and without spacer were not separately delineated. Additionally, the MIRAGE study was conducted using a low-field, instead of high-field, MR-LINAC. Thus, our study provided valuable insights into longitudinal clinical outcomes of high-field MRgSBRT using primarily HA rectal spacers, despite a number of study limitations.

Our preliminary results reconfirmed the low risk and low complication rate associated with rectal spacer insertion in localized prostate cancer patients, as well as improved dosimetry in treatment plans due to the increased prostate–rectum distance, similar to many previous studies [7, 8, 20, 23]. Dosimetry in terms of target coverage and rectal sparing was significantly better in the spacer cohort than in the non-spacer cohort. Notably, the non-spacer cohort had a larger balloon volume (80–90 mL) than the spacer cohort (40–50 mL), resulting in a significantly larger rectum volume. While the dosimetric impact of the rectal balloon on the rectal wall is inconclusive in the literature, large balloon volumes are reportedly beneficial for rectal dose sparing [26]. Therefore, the dosimetric advantage of the spacer on the rectum might not have been compromised by the balloon volume difference. Although the majority of our cohort received HA spacer, in contrast to previous studies using hydrogel spacer, our findings were generally consistent with previous reports [10, 23, 27] in terms of very low incidence of clinician-reported rectal and GI toxicities. There were no G2-or-higher GI toxicities throughout the follow-up, which may represent an improvement over some previous spacer studies in the non-MR-guided SBRT setting [28, 29]. Our GI toxicities were also comparable or even lower than those in the spacer arm of a phase III study of hypofractionated RT using an HA spacer [10], possibly because of MRI guidance. Our acute GI toxicity data were consistent with those reported by an Italian group using the same MR-LINAC facility for MRgSBRT, in which no G2-or-above GI toxicities were observed in 10 patients with a 3-month follow-up [17, 20]. We suspect that the favorable GI toxicity performance is attributable to the excellent visibility of spacer and soft tissues on planning and daily MR images, and the opportunity for daily online plan adaptation, despite the differences in configurations between rectal spacer + rectal balloon versus rectal spacer alone. Notably, compared with the Italian study, our sample size (*n* = 34 vs. 10) was larger, included high-risk patients (*n* = 15, ~ 44%), and had a longer follow-up (median: ~ 13 months vs. 5 months). Moreover, the GI status did not deteriorate, even among our high-risk patients who underwent concomitant whole-pelvic RT and/or DIL boosting along with irradiation to the prostate gland, highlighting the potential clinical value of rectal spacers in such patients. The patients also reported favorable GI QOL scores. The EPIC bowel domain summary scores were similar to those reported by Alongi et al. [20]. The excellent bowel function and bother scores in all follow-up phases also suggested the potential clinical efficacy of rectal spacers for maintaining bowel QOL scores.

Despite the encouraging preliminary results observed in this study, it is worth pointing out that our study did not formally address a fundamental question of whether the potential advantages of spacer insertion are eclipsed by the inherent benefits of MRgRT in terms of superior image contrast, online adaptation, and advanced motion management of MRI guidance. In this study, we postulated that both MRI guidance and spacer insertion led to GI toxicity reduction. However, it is worth investigating the feasibility of avoiding the invasive spacer insertion in MRgRT while maintaining GI toxicity reduction in the long term. It is also worth exploring whether MRgRT plus spacer insertion would further improve GI toxicity compared with using either of these approaches alone. Relevant studies are ongoing in our center.

This study has several limitations. First, the single-center, single-arm, non-randomized study design with a small sample size considerably limited the generalizability. The inability to capture all potential confounding variables should also be noted. Second, dosimetry and clinical outcomes were retrospectively compared to the literature and our own previous data, which might have compromised the statistical strength to some extent. Third, this study might be liable to selection bias because it only included patients who were willing to undergo spacer placement. Fourth, the included patients had different risk levels, necessitating heterogeneity in SBRT and ADT. Some patients underwent irradiation to the prostate only, whereas high-risk patients also underwent dose boosting to intraprostatic lesions and concomitant whole-pelvis irradiation. Fifth, the follow-up duration was relatively short, limiting information on long-term toxicities, QOL, and biochemical recurrence-free survival. Finally, the completeness of QOL data was compromised by a number of patients who failed to submit EPIC questionnaires on schedule, or left some items (e.g., in the sexual domain) blank during follow-up. This substantially affected the usable sample size and statistical analysis of the PRO assessment. Meanwhile, these missing data were not missing completely at random (MCAR), and no imputation was applied for compensation. Thus, the results of the QOL analysis might have been biased and prone to a false discovery rate, which should be further validated by a larger sample size. Multicenter, two-arm randomized clinical trials should be developed in the future.

## Conclusions

The present study prospectively explored the utilization of rectal spacer in adaptive MRgSBRT for localized prostate cancer patients and reported clinical PROs and CROs within a median follow-up of 13 months. Rectal spacer insertion had a low complication rate, and the rectal dose exposure was significantly reduced by the increase in rectum–prostate distance. Minimal G1 and no G2 rectal toxicities were found throughout the follow-up period. PROs in bowel functions and bother remained mostly constant throughout the entire follow-up, except for a slight drop in the acute phase. These encouraging preliminary results strongly suggest the safety, dosimetric merit, minimal toxicity, and patient satisfaction with the utilization of rectal spacer in MRgSBRT. Randomized trials with a larger sample size and a longer follow-up are warranted for further validation.

## Supplementary Information

Below is the link to the electronic supplementary material.

Supplementary file1 (DOCX 17 KB)

## Data Availability

The data that support the findings of this study are available from the corresponding author upon reasonable request.

## Abbreviations

ADT: Androgen-deprivation therapy
ATP: Adapt-to-position
ATS: Adapt-to-shape
CRO: Clinician-reported outcome
CTV: Clinical target volume
CTCAE: Common Terminology Criteria for Adverse Events
CT: Computed tomography
DIL: Dominant intraprostatic lesion
ECOG: Eastern Cooperative Oncology Group
EPIC: Expanded Prostate Cancer Index Composite
GI: Gastrointestinal
GU: Genitourinary
HA: Hyaluronic acid
MR: Magnetic resonance
MRgSBRT: Stereotactic body radiation therapy with online guidance with magnetic resonance imaging
MR-LINAC: Integrated MRI and linear accelerator
MRI: Magnetic resonance imaging
OAR: Organ-at-risk
PRO: Patient-reported outcome
PET: Positron emission tomography
PTV: Planning target volume
PSA: Prostate-specific antigen
PSMA: Prostate-specific membrane antigen
QOL: Quality of life
RT: Radiation therapy
SD: Standard deviation
SBRT: Stereotactic body radiation therapy
TSE: Turbo spin echo
