# Supplementary material for: One-year clinical outcomes of MR-guided stereotactic body radiation therapy with rectal spacer for patients with localized prostate cancer
Source: World J Urol. 2024 Feb 23;42(1):97. doi: 10.1007/s00345-024-04784-x (PMC10891188; doi:10.1007/s00345-024-04784-x)
Supplement: Supplementary file 1 — Supplementary file1 (DOCX 17 KB) [file 345_2024_4784_MOESM1_ESM.docx]

**Supplementary Table 1**. Dosimetry of target organs and OARs in treatment plans with and without rectal spacer insertion

|  | | **Spacer**  Median (range) | **Non-spacer**  Median (range) | ***P* value**  (Wilcoxon signed-rank) |
| --- | --- | --- | --- | --- |
| Age, years (range) | | 70 (58–82) | 74 (57–90) | 0.10 |
| Risk group,  no. of patients | Low | 4 | 3 | 0.71 (chi-square test) |
|  | Favorable Intermediate | 12 | 8 |  |
|  | Unfavorable Intermediate | 3 | 4 |  |
|  | High | 12 | 13 |  |
|  | Very High | 3 | 6 |  |
| Medical and surgical comorbidity | Diabetes | 3 | 2 | All p>0.05 (chi-square test) |
|  | Hypertension | 22 | 21 |  |
|  | Ischemic heart disease | 6 | 8 |  |
|  | Hemorrhoid | 0 | 1 |  |
|  | Prior abdominal or pelvic surgery | 3 | 4 |  |
|  | Use of anti-coagulants | 6 | 6 |  |
| Prostate volume, cc (range) | | 39.3 (14.6–113) | 35.4 (13.9–180.4) | 0.37 |
| *PTV* | V105–116% <1 cc (cc) | 0.5 (0–11.8) | 0.5 (0–6.3) | 0.21 |
|  | V100% >95% (%) | 98.6 (93.4–99.8) | 97.8 (69.6–99.7) | 0.03* |
| Rectum volume, cc (range) | | 95.5 (55.5–173.8) | 149.2 (105.3–198) | <1e-5* |
| *Rectum* | V105% <0.03 cc (cc) | 0 (0–0.5) | 0 (0–2.4) | 0.00061* |
|  | V95% <3 cc (cc) | 0.7 (0–4.6) | 4.9 (0–12.5) | <1e-5* |
|  | V90% <10% (%) | 1.9 (0–9.1) | 4.9 (1.4–11.1) | 0.00011* |
|  | V80% <20% (%) | 4.7 (0–13.5) | 8.3 (2.9–17.1) | 0.0011* |
|  | V50% <50% (%) | 25.7 (3.8–43.1) | 23.7 (10.7–42.4) | 0.56 |
| Bladder volume, cc (range) | | 160.6 (107.2–235.8) | 158.9 (81.8–237.8) | 0.78 |
| *Bladder* | V105% <1 cc (cc) | 0 (0–2.3) | 0 (0–0.4) | 0.45 |
|  | V92.5% <5 cc (cc) | 6.6 (1.5–14.6) | 6.6 (0.5–16.2) | 0.30 |
|  | V90% <10% (%) | 6.3 (1.4–13.8) | 5.6 (1.0–18.3) | 0.78 |
|  | V50% <50% (%) | 38.8 (10.27–74.69) | 38.3 (9.3–57.9) | 0.87 |
|  | V45% <60% (%) | 50 (11.6–83.3) | 49.7 (12.2–64.4) | 0.81 |
| *Femoral Head* | V20Gy <10 cc (cc) | 0.2 (0–20.6) | 1.8 (0–43.6) | 0.45 |
|  | Dmax <30 Gy (Gy) | 21.2 (13.9–28.2) | 21.8 (0–28.2) | 0.83 |
| Penile bulb volume, cc (range) | | 3.3 (0.7–8.3) | 3.6 (1.3–14.2) | 0.90 |
| *Penile Bulb* | D2% <28.5 Gy (Gy) | 26.2 (8.2–48.0) | 28.7 (3.8–42) | 0.81 |
|  | V20Gy <3 cc (cc) | 0.3 (0–2.0) | 0.4 (0–7.3) | 0.62 |

*Indicates items with statistical significance (*p*<0.05)
